# Use of arteriography for the initial evaluation of patients with intermittent lower limb claudication

**DOI:** 10.1590/S1516-31802001000200004

**Published:** 2001-03-02

**Authors:** Nelson Wolosker, Ruben Ayzin Rosoky, Kenji Nishinari, Lívio Nakano

**Keywords:** Angiography, Atherosclerosis, Complications, Risk factors, Follow-up studies, Angiografia, Aterosclerose, Complicações, Fatores de risco

## Abstract

**CONTEXT:**

Many patients with intermittent claudication continue to be forwarded to the vascular surgeon for initial evaluation after arteriography has already been accomplished.

**OBJECTIVE:**

The main objective of this work was to analyze the usefulness and the need for this procedure.

**TYPE OF STUDY:**

Retrospective study.

**SETTING:**

The patients were divided into two groups: Group 1, with the arteriography already performed and Group 2 without the initial arteriography.

**PARTICIPANTS:**

One hundred patients with intermittent claudication were retrospectively studied. Other specialists had forwarded them for the first evaluation of intermittent claudication, without any previous treatment.

**MAIN MEASUREMENTS:**

All patients were treated clinically for at least a 6-month period. The total number of arteriographies performed in the two groups was compared and the need and usefulness of the initial arteriography (of Group 1) was also analyzed.

**RESULTS:**

The evolution was similar for both groups. The total number of arteriographies was significantly higher in Group 1 (Group 1 with 53 arteriographies vs. Group 2 with 7 arteriographies). For this group, it was found that arteriography was only useful in five cases (10%), because the surgeries were based on their findings. However, even in those cases, no need for arteriography was observed, as the procedure could have been performed at the time of surgical indication.

**CONCLUSION:**

Thereare no indications for arteriography in the early evaluation of patients with intermittent claudication, because it does not modify the initial therapy, independent of its result. In cases where surgical treatment is indicated, this procedure should only be performed prior to surgery.

## RESUMO

**CONTEXTO:**

Muitos pacientes com claudicação intermitente de membros inferiores (claudicação intermitente) são encaminhados para avaliação inicial do cirurgião vascular com arteriografia já realizada.

**OBJETIVO:**

Analisar a utilidade e a necessidade dessa arteriografia.

**TIPO DE ESTUDO:**

Estudo retrospectivo.

**LOCAL:**

Hospital das Clínicas da Universidade de São Paulo.

**PARTICIPANTES:**

Foram estudados retrospectivamente 100 pacientes com claudicação intermitente sem tratamento anterior, encaminhados por outros especialistas para avaliação inicial no Serviço de Cirurgia Vascular

**VARIÁVEIS ESTUDADAS:**

Os pacientes foram divididos em dois grupos: Grupo 1, com arteriografia realizada antes da avaliação inicial e Grupo 2 sem arteriografia. Analisamos a utilidade e a necessidade da arteriografia inicial e, também, comparamos o número total de arteriografia realizadas nos dois grupos.

**RESULTADOS:**

A evolução clínica nos dois grupos foi semelhante. O número total de arteriografia foi significativamente maior no Grupo 1 (Grupo 1 - 53 arteriografias X Grupo 2 - 7). Nesse grupo, somente em cinco casos (10%) as arteriografias realizadas antes da avaliação inicial foram úteis, pois as operações foram baseadas em seus achados. Porém, mesmo nesses casos, não houve a necessidade dessa arteriografia, pois esse procedimento poderia ter sido realizado na época da indicação da operação.

**CONCLUSÃO:**

Concluiu-se que não existe indicação para a realização da arteriografia antes da avaliação inicial dos pacientes com claudicação intermitente, pois não modifica a conduta inicial, independente de seu resultado, e, nos casos em que o tratamento cirúrgico é indicado, esse exame deve ser realizado próximo a operação.

## INTRODUCTION

Intermittent claudication is usually the first clinical symptom of peripheral chronic arterial disease. In many patients, it can progress with severe limitations on walking long distances, and on social, work and leisure activities. The intermittent claudication diagnosis is based on history, physical examination and non-invasive tests: the ankle-brachial index and treadmill test with Doppler.^1,2^ A more accurate anatomical and functional diagnosis can be done with the duplex scan.^3^

The initial treatment is always conservative,^1^ independent of the degree or of the level of arterial obstruction. Detailed studies on the arterial lesions are not necessary at this stage, because they will not change the type of approach. Despite this, many patients are sent to a vascular surgeon for initial evaluation, even after the angiography has already been performed.

Surgery is only proposed for patients in two situations:

- In cases of disabling intermittent claudication, when the feasible walking distance before the onset of intermittent claudication is too short, limiting habitual activities and the patient does not show any improvement after six months of clinical treatment.
- When the patient shows critical ischemia (rest pain or ischemic trophic lesions), there is a high risk of limb loss, if the patient remains on conservative treatment.

Nowadays, arteriography is considered the best guideline for choosing the most appropriate surgical procedure.

The main objective of this work was to analyze the usefulness and the need for arteriography for the initial treatment of patients with intermittent claudication.

The costs of diagnostic examinations (angiography and duplex-scan) are also compared between patients with or without arteriography done at the time of the initial examination.

## METHODS

One hundred patients with intermittent claudication were retrospectively surveyed for a prospective study (72 males and 28 females with a mean age of 56 years). They were forwarded to the outpatient service of the Department of Vascular Surgery of the university clinical hospital of São Paulo Medical School by other specialists, without having undergone any previous treatment.

Patients were divided into two groups as they came in to a vascular surgeon: Group 1, consisting of 50 patients with arteriography already performed; Group 2, consisting of patients without arteriography (the last 50 consecutive patients in the claudication study, on which the examination had not been performed).

All patients were evaluated and submitted to conservative treatment, which consisted of controlling the risk factors and physical training, represented by one-hour-long walks daily over a minimum period of 6 months. The patients were evaluated via periodical medical follow-up. The average follow-up was 31 months.

Patients with clinical improvement or stability were maintained on conservative treatment.

Surgical treatment was proposed for all patients with disabling intermittent claudication (when the walking distance limits their daily activities), but some of them chose to continue the conservative treatment after discussing the surgical risks and possible complications.^4,5^

All patients with critical ischemia were submitted to surgical treatment, since this situation implies a high risk of limb loss.

Clinical characteristics and development were compared between groups, as well as the number of arteriographies performed in each group. For Group 1, the usefulness and the need for the initial arteriography in the treatment of these patients were analyzed.

Usefulness was defined as the condition in which the examination was utilized at any time during treatment, to reach a protocol decision as required, and the need was defined as whenever the examination was essential for treatment of the patient.

Most of the arteriographies in Group 1 were done in other Hospitals. To calculate the costs of the exams, the prices of each one in our hospital were considered.

The average cost of each arteriography was US$ 1000 and each duplex scan was US$ 250.

### Statistical analysis

Statistical comparisons between variables were performed using Fisher's exact test and chisquared analysis. Results were considered significant when P < 0.05.

## RESULTS

The clinical features are presented in Table 1. The same clinical status was verified in both groups.

**Table 1:** Clinical features

Features	Group 1	Group 2
Age, years.	57	55
Gender	37m 13f	35m 15f
Smoking, %	88	90
Hypertension, %	62	58
Diabetes Mellitus, %	20	18
Hyperlipidemia, %	22	18

m = male; f = female.

The clinical evolution is presented in Table 2. There was no statistical difference between the clinical evolution between the groups.

**Table 2:** Clinical evolution

Clinical evolution	Group 1	Group 2	Total
Improvement or Stability	35 (70%)	37 (74%)	72 (72%)
Worsening	15 (30%)	13 (26%)	28 (28%)
**Total**	**50**	**50**	**100**

For Group 1, the condition of 15 patients worsened. Surgery was proposed for all of these patients, but six patients with disabling intermittent claudication chose to continue with clinical treatment alone. The other nine patients were submitted to surgery (three for critical ischemia and six for worsening of intermittent claudication). The surgical procedures were: six angioplasties of the iliac artery, one aortobifemoral graft, one bilateral lumbar sympathectomy and one primary transfemoral amputation.

In this group, except for the patient who needed a primary amputation, a duplex scan was used to search for anatomical similarities with the initial arteriography, trying to avoid the need for a more invasive examination. However, for three patients, anatomical changes identified on the duplex-scan indicated a new arteriography for surgical planning. One patient had a massive myocardial infarct with hypotension, suffering critical ischemia of the left limb. Due to the high surgical risk, this patient was submitted to a primary amputation.

The total number of arteriographies performed in Group 1 was fifty-three. Fifty arteriographies were done before the initial evaluation of our service. Three more arteriographies were necessary for evaluation of any anatomical changes before a surgical procedure.

Analyzing each case in Group 1, the need and usefulness of each arteriography were checked. There was no need for the initial arteriography, under different conditions, and only 10% of the arteriographies were useful during treatment.

For Group 2, the number of arteriographies performed was seven (P < 0.01).

The number of surgeries was similar in both groups: seven in Group 1 and nine in Group 2.

In Group 2, the condition of 13 patients worsened. Surgery was proposed for all of them, but six patients with disabling intermittent claudication chose to continue with only clinical treatment. The other seven patients were submitted to surgery (four with critical ischemia and three with worsening of intermittent claudication). The surgical procedures were: three angioplasties of the iliac artery, two aortic-bifemoral bypasses, one femoral-popliteal bypass and one iliac-femoral bypass. Only seven arteriographies were done for surgical planning.

Table 3 shows the costs of these complementary studies in each group.

**Table 3:** Costs (in US$) of laboratory examinations ordered for all patients

Examinations	Group 1	Group 2
AG (US$)	(50+3) x 1000	7 × 1000
Duplex (US$)	8 × 250	0
**Total (US$)**	**55,000**	**7,000**

AG - arteriography.

## DISCUSSION

Intermittent claudication of the lower limbs usually hinders the patient's habitual activities and the objective of the treatment is to improve the quality of life.^6^

Initial treatment is always conservative, because intermittent claudication has a good evolution. Most of the patients have improvement or stability of the clinical manifestations,^7,**8**^ with low risk of amputation. In our service, the patients are submitted to conservative treatment for at least six months: unsupervised physical exercises, with one-hour daily walking^9^ and control of the main atherosclerotic risk factors (smoking,^10^ hypertension,^11^ diabetes^12^ and hyperlipidemia^13^).

Surgical treatment is only indicated when clinical treatment is not effective (critical ischemia or limiting intermittent claudication).^14,15^ All of the patients with critical ischemia were submitted to surgery but some of the patients with no clinical improvement refused the operation because of the surgical risks.

Arteriography is still the best examination for cases requiring surgical treatment, because it supplies precise anatomical information, allowing the best operative technique to be used with a narrower margin of error.^16^

Notwithstanding technical innovations, modern guide-wires, low profile sheets, resistant catheters, contrasts with low osmolarity and the digital equipment, arteriography presents considerable morbidity ranging from 0.8%^17^ to 7%^18^ and mortality rates ranging from 0.03%^19^ to 0.7%.^18^

In this study, only 5 arteriographies (10%) accomplished prior to the clinical treatment were of any use for management definition. However, these patients needed an additional evaluation with the duplex scan to confirm anatomical similarities between the times of surgical indication and arteriography. Even in these cases, there was no need, because all of them could have been performed at the time of the surgical indication.

In three patients submitted to surgery in Group 1, physical examination and duplex scan showed differences. For this reason, the initial arteriography was considered inappropriate for surgical programming and a new one was performed. Therefore, in 33% of the patients submitted to surgical treatment, the arteriography was performed twice, increasing risks and costs. In these cases, the need and usefulness was considered null.

One patient was submitted to primary amputation due to critical ischemia of the leg, concomitant to acute myocardial infarction with severe clinical consequences, that impaired any possibility of arterial restoration. In this case there was no usefulness or need for the initial arteriography.

In spite of the fact that the patients did not develop any complications related to the arteriography, they had been submitted to an invasive exam, which was useful in only 10% of the cases and was not needed. The costs of complementary exams were eight times higher in Group 1 compared to Group 2, mostly as a result of the arteriographies done in the initial evaluations of Group 1 patients.

## CONCLUSION

It was concluded that there is no indication for arteriography for patients who, at initial evaluation, present with intermittent claudication because, independent of the outcome, it does not modify early management. For cases in which surgical treatment is the procedure of choice, this examination should only be performed prior to surgery, for its planning.
